# Auditory Beat Stimulation Modulates Memory-Related Single-Neuron Activity in the Human Medial Temporal Lobe

**DOI:** 10.3390/brainsci11030364

**Published:** 2021-03-12

**Authors:** Marlene Derner, Leila Chaieb, Gert Dehnen, Thomas P. Reber, Valeri Borger, Rainer Surges, Bernhard P. Staresina, Florian Mormann, Juergen Fell

**Affiliations:** 1 Department of Epileptology, Venusberg-Campus 1, University Hospital Bonn, 53127 Bonn, Germany; marlene.derner@ukbonn.de (M.D.); leila.chaieb@ukbonn.de (L.C.); gert.dehnen@rub.de (G.D.); treber@live.com (T.P.R.); rainer.surges@ukbonn.de (R.S.); florian.mormann@ukbonn.de (F.M.); 2 Faculty of Psychology, Swiss Distance University Institute, Ueberlandstr. 12, 3900 Brig, Switzerland; 3 Department of Neurosurgery, Venusberg-Campus 1, University Hospital Bonn, 53127 Bonn, Germany; valeri.borger@ukbonn.de; 4 Centre for Human Brain Health, School of Psychology, University of Birmingham, Birmingham B15 2TT, UK; b.staresina@bham.ac.uk

**Keywords:** long-term memory, microwire recordings, item recognition, source recognition, binaural beats, monaural beats

## Abstract

Auditory beats are amplitude-modulated signals (monaural beats) or signals that subjectively cause the perception of an amplitude modulation (binaural beats). We investigated the effects of monaural and binaural 5 Hz beat stimulation on neural activity and memory performance in neurosurgical patients performing an associative recognition task. Previously, we had reported that these beat stimulation conditions modulated memory performance in opposite directions. Here, we analyzed data from a patient subgroup, in which microwires were implanted in the amygdala, hippocampus, entorhinal cortex and parahippocampal cortex. We identified neurons responding with firing rate changes to binaural versus monaural 5 Hz beat stimulation. In these neurons, we correlated the differences in firing rates for binaural versus monaural beats to the memory-related differences for remembered versus forgotten items and associations. In the left hemisphere, we detected statistically significant negative correlations between firing rate differences for binaural versus monaural beats and remembered versus forgotten items/associations. Importantly, such negative correlations were also observed between beat stimulation-related firing rate differences in the pre-stimulus window and memory-related firing rate differences in the post-stimulus windows. In line with concepts of homeostatic plasticity, our findings suggest that beat stimulation is linked to memory performance via shifting baseline firing levels.

## 1. Introduction

Auditory beat stimulation is a non-invasive brain stimulation technique, for which effects on anxiety and cognition including memory have been reported (for overviews, see, e.g., [1,2]). Auditory beats are amplitude-modulated tones with modulation frequencies in the range of typical electroencephalographic (EEG) rhythms. For instance, beat signals can be constructed by superposing two sine waves with nearby frequencies. Beat stimulation is either applied by presenting amplitude-modulated beat signals to one ear or both ears (monaural beats), or by presenting the original sine waves separately to each ear (binaural beats). In this latter, more frequently investigated case, beat perception results from the responses of phase-sensitive brain stem neurons of the superior olivary complex [3]. In prior investigations, it has been shown that monaural and binaural beats have a different impact on EEG power, phase patterns and phase synchronization (e.g., [4,5,6]). Phase synchronization and other phase-related mechanisms play an important role in long-term memory processing (e.g., [7]); therefore, differential effects of monaural and binaural beats on long-term memory performance are to be expected.

Based on intracranial EEG (iEEG) recordings in presurgical epilepsy patients, in a previous study we demonstrated that monaural and binaural beat stimulation caused changes in iEEG power and phase synchronization [4]. These effects were most prominent at a modulation frequency of 5 Hz and were observed in temporal regions, as well as in mediotemporal structures (e.g., rhinal cortex and hippocampus), which play a crucial role in long-term memory (e.g., [8]). More specifically, we found an increase in temporo-lateral phase synchronization for 5 Hz binaural beats and a decrease in mediotemporal phase synchronization for 5 Hz monaural beats. Due to the role of phase synchronization in long-term memory, we hypothesized that binaural versus monaural 5 Hz beats may be related to increased versus decreased memory performance. In a subsequent study, we therefore investigated the impact of monaural and binaural 5 Hz beat stimulation on long-term memory performance in a task comprising the learning and recognition of words and associated colors or scenes [5]. We observed a linear effect, indicating that compared to control stimulation, binaural 5 Hz beats increased and monaural 5 Hz beats decreased item memory for words, as well as source memory for associated information. These behavioral effects corresponded to reverse iEEG phase shifts within the rhinal cortex for binaural versus monaural beat stimulation. iEEG phases likely influence neural membrane potentials and firing thresholds, and thereby control whether neural activity—in this case memory-related activity—occurs within the required time window or not. The aim of the present study was to investigate the open question, whether monaural and binaural 5 Hz beat stimulation cause changes in neural firing, which are related to the modulation of memory performance.

To answer this question, we analyzed the activity of single neurons recorded from the amygdala, hippocampus, entorhinal cortex, and parahippocampal cortex in a subgroup of the previously investigated patients ([5]; see also [9]). Following spike detection and sorting, we identified neurons responding with firing rate changes to binaural versus monaural 5 Hz beat stimulation. In these neurons, we correlated the differences in firing rates for binaural versus monaural beats to the memory-related differences for remembered versus forgotten items/associations. Specifically, we assessed two alternative possibilities. Both are based on the assumption that remembering and forgetting are dependent upon the difference between memory-related and baseline firing rates: (i) beat- and memory-related firing rate changes are positively correlated: this would mean that beat stimulation-related modulations of firing rates are linked to memory performance via directly adding to memory-related firing rate changes; (ii) beat- and memory-related firing rate changes are negatively correlated. This would imply that beat stimulation is linked to memory performance via shifting baseline firing levels and thereby modulating differences between memory-related and baseline firing rates. The latter possibility is in line with the Bienenstock–Cooper–Munro (BCM) learning rule [10]. The BCM rule proposes a sliding threshold for the induction of either long-term potentiation or long-term depression in response to alterations in neural activity. More specifically, according to this rule, high/low levels of previous neuronal activity favor synaptic depression/facilitation by increasing/decreasing the crossover threshold between long-term potentiation and depression. The idea that this mechanism enables continuous adaptation of synaptic strengths to a physiological range is supported by empirical evidence (see e.g., [11]). Furthermore, we hypothesized that correlations between beat- and memory-related firing rate changes may in particular be observed in the left hemisphere because of its specialization for language processing (e.g., [12]).

## 2. Materials and Methods

### 2.1. Experimental Design

#### 2.1.1. Participants

Recordings from five presurgical epilepsy patients (3 females (age: 26/42/47 years), 2 males (age: 36/48 years)) with implanted microwires were analyzed. These patients represent a subset of a group of 15 patients (microwires had only been implanted in this subset), for whom results from macro-electrode recordings were previously reported [5]. All patients gave informed written consent, and the study was conducted according to the Declaration of Helsinki, and approved by the ethics committee of the Medical Faculty of the University of Bonn (ethical approval code: 401/16).

#### 2.1.2. Experimental Paradigm

**Figure 1:**
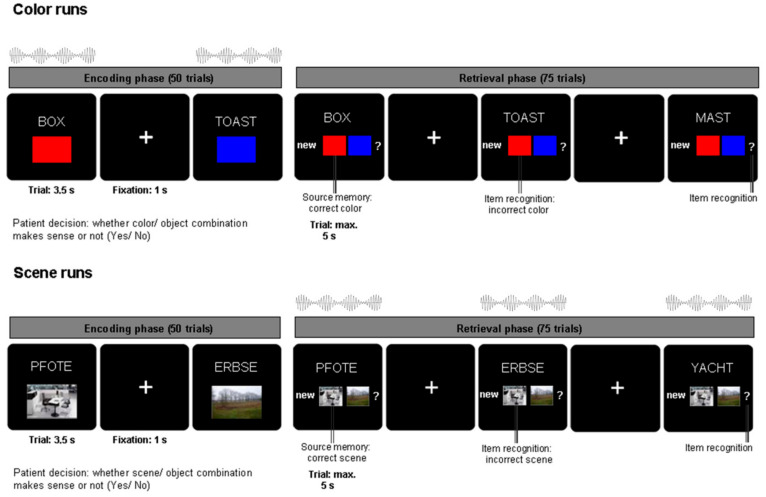
**Experimental paradigm:** Schematic depiction of the associative memory paradigm and the beat stimulation interventions (see Methods).

Subjects were asked to perform an associative memory task (see Figure 1) as described in an earlier study (e.g., [13]). During the encoding phase of the task, 50 German nouns (per run; on each run different nouns) were presented together with a color patch (red/blue) or an image of a scene (office/nature) for 3.5 s each. During a jittered inter-trial interval of 700–1300 ms (mean = 1000 ms) a fixation cross was presented. Subjects were asked to indicate with a button press whether the association between the color/scene and noun was plausible or not. The retrieval phase started after a 1 min break. The 50 nouns previously presented during the encoding phase were shown together with 25 novel, previously unstudied nouns. The response options were (1) “new”, (2) the two possible color/scene sources (indicating an “old” response with source memory), and (3) a question mark (indicating an “old” response without source memory). Each response trial was displayed for a maximum of 5 s. In each experimental run, only one source category (either color or scene) was used.

Across six experimental runs, auditory beat stimuli or control tones were presented to the subjects either during the encoding phase (for color source runs only) or the retrieval phase (for scene source runs only) (see also [5]). The stimulation conditions were: binaural beats (5 Hz), monaural beats (5 Hz), control tone (220 Hz—no beat). Beat stimulation was presented at stimulus onset for the duration of each trial (encoding: 3.5 s, retrieval: 5 s). Auditory beats were composed of two sine waves with frequencies of 217.5 Hz and 222.5 Hz. Monaural beats resulting from the physical superposition of the two sine waves were presented to both ears simultaneously. In the case of binaural beats, one sine wave (e.g., 217.5 Hz) was presented to one ear, while the other sine wave (e.g., 222.5 Hz) was presented to the opposite ear. Each of the five subjects completed six runs each comprising 50 encoding trials and 75 retrieval trials (i.e., 300 encoding and 450 retrieval trials per subject). The order of beat stimulation conditions across the six runs had been counterbalanced within the original group of 15 patients.

#### 2.1.3. Data Recording and Preprocessing

Action potential recordings were obtained from a bundle of nine microwires (eight high-impedance recording electrodes, one low-impedance reference; Ad-Tech (Medical Instrument Corporation, Oak Creek, WI, USA)) protruding from the end of each depth electrode targeting the hippocampus, entorhinal cortex, amygdala and parahippocampal cortex. The differential signal from the microwires was amplified using a Neuralynx ATLAS system (Neuralynx, Bozeman, MT, USA), filtered between 0.1 and 9000 Hz, and sampled at 32 kHz. These recordings were stored digitally for further analysis. The number of recording microwires per patient ranged from 80 to 96. Signals were band-pass filtered between 300 and 3000 Hz. Spike detection and sorting was performed using the Combinato software package [14]. After automated sorting using standard parameters, clusters in every channel were manually adjusted based on cluster shape, cross correlograms, and other features provided by the Combinato package. Sorted units were classified as single units, multi-units, or artifacts (Supplementary Figures S1–S3) based on spike shape and variance, ratio between spike peak value and noise level, the inter-spike interval distribution of each cluster, and presence of a refractory period for the single units [15,16].

Anatomical localization of microwires was determined based on the post-implantation CT scan co-registered to the pre-implantation MRI scan, both normalized to MNI (Montreal Neurological Institute) space with SPM12. In detail, firstly, the end of the corresponding depth electrode was visually identified. Then, microwire loci were inferred from a 3 mm-radius sphere placed 4 mm medial to the electrode tip [17]. Anatomical regions within the MTL were delineated visually according to segmentation protocols described by Insausti et al. [18] and Pruessner et al. [19,20]. Only data from microwires unambiguously localized within amygdala, hippocampus, entorhinal cortex or parahippocampal cortex were kept for further analysis.

### 2.2. Statistical Analysis

#### 2.2.1. Evaluation of Firing Rates

Firing rates for each trial were calculated for four non-overlapping 500 ms intervals from 0 ms to 2000 ms, relative to stimulus onset. Only units with a minimal average firing rate of 2 Hz across trials in at least one interval in one of the three stimulation conditions (in encoding or retrieval) were kept for further analysis, leaving 180 units (left side: 112, right side: 68; 95 single units, 85 multi units; 166 units removed due to 2 Hz criterion) from four brain regions (49 in the amygdala (27%; left: 31, right: 18), 32 in the hippocampus (18%; left: 13, right: 19), 63 in the entorhinal cortex (35%; left: 49, right: 14), 36 in the parahippocampal cortex (20%; left: 19, right: 17)). The contributions of the five participants to the 180 units were: p1 (45); p2 (44); p3 (16); p4 (64); p5 (11). We applied a 2 Hz cutoff threshold, because including units with a lower firing rate would impair the statistical robustness of our analyses and the role of sparsely firing units in neural coding is currently unclear. 

#### 2.2.2. Relationship between Beat Stimulation and Memory Performance: Behavioral Data

In a previous study [5], we reported enhanced item and source memory for binaural compared to monaural beats during both encoding and retrieval in a group of 15 patients. For the present study, a subgroup of five patients in whom microelectrode data had been recorded, were analyzed. To evaluate the influence of beat stimulation on item and source memory in this subgroup, we conducted two-way repeated-measures ANOVAs (factors: beat condition (binaural, monaural); task phase (encoding, retrieval)) with the percentage of hits and percentage of correct source decisions as dependent variables. 

#### 2.2.3. Relationship between Beat Stimulation and Memory Performance: Neural Data

To investigate the interrelation between the behavioral effect of beat stimulation on memory and neural activity patterns [5], we first identified units which showed different firing rates for binaural vs. monaural beat stimulation separately during encoding and retrieval, as well as separately for the left and right hemispheres (Wilcoxon tests for each of the four 500 ms time windows; Bonferroni-corrected threshold of *p* ≤ 0.0125). Binomial tests with a probability 0.05 (corresponding to the alpha level of 5%) were conducted to test if the number of units showing a significant contrast was significantly higher than expected by chance. The significance of overlap between units related to different contrasts was calculated accordingly as portion_class1*portion_class2 (instead of 5% for single classes).

For these units, we then extracted firing rates for each time window during encoding and retrieval, depending on whether items (words) or sources (colors/scenes) were remembered or forgotten. This means that item-related responses were classified as (later) remembered versus (later) forgotten if old words were correctly classified as old versus wrongly classified as new. Source-related responses were classified as (later) remembered versus (later) forgotten if colors/scene were correctly identified versus wrongly assigned or unknown. We then calculated the normalized firing rate (fr) differences (Diff) related to beat stimulation, as well as related to memory. Normalization was performed by dividing differences of firing rates for the 500 ms intervals by the sum of firing rates during the whole 2 s interval: Diff_binaural,monaural_ = (fr(binaural) − fr(monaural))/(fr_[0;2s]_(binaural) + fr_[0;2s]_ (monaural)); Diff_remembered,forgotten_ = (fr(remembered) − fr(forgotten))/(fr_[0;2s]_(remembered) + fr_[0;2s]_(forgotten)).

Firing rate differences were normalized to exclude trivial influences of firing rate magnitude. Memory-related differences were calculated across all experimental runs in order to achieve robust estimates. As an additional analysis, memory-related differences were extracted only from the runs during which beat stimulation had been applied (encoding/color and retrieval/scene runs). Finally, for each time window during encoding and retrieval, Pearson’s correlations between normalized beat stimulation-related and memory-related firing rate differences were calculated. Correlation values with a *p*-value below 0.0125 (Bonferroni-correction for four time windows) were considered significant.

#### 2.2.4. Data and Code Availability

In accordance with the ethics approval given by the ethics committee of the Medical Faculty of the University of Bonn and the guidelines of the German Research Foundation, pooled spiking data and program codes have been made publicly available to researchers on the Github Online Repository, https://github.com/LeilaChaieb/Fell_Workspace (accessed on 3 December 2020). Further queries should be addressed directly to the corresponding author via email.

## 3. Results

### 3.1. Behavioral Data

Across the group of five subjects, the percentage of hits (correct old decisions) for color/scene runs was: 86/83% ± 5/4% (mean ± s.e.m). The percentage of false alarms (old decisions in the case of new words) was: 19/19% ± 5/4%. The probability measure hits minus false alarms revealed that recognition memory was significantly above chance: 67/64% ± 4/6% (*t*-tests, both *p* < 0.001, t_4_ = 17.62/10.99). The percentages of correct, incorrect, and unsure source decisions were: 63/68% ± 3/6%, 24/23% ± 7/8% and 12/9% ± 6/5%, respectively. Probability for source recognition (correct minus incorrect source decisions) was also significantly above chance: 39/45% ± 8/13% (*p* = 0.009/0.028, t_4_ = 4.68/3.37).

### 3.2. Relationship between Beat Stimulation and Memory Performance: Behavioral Data

**Figure 2:**
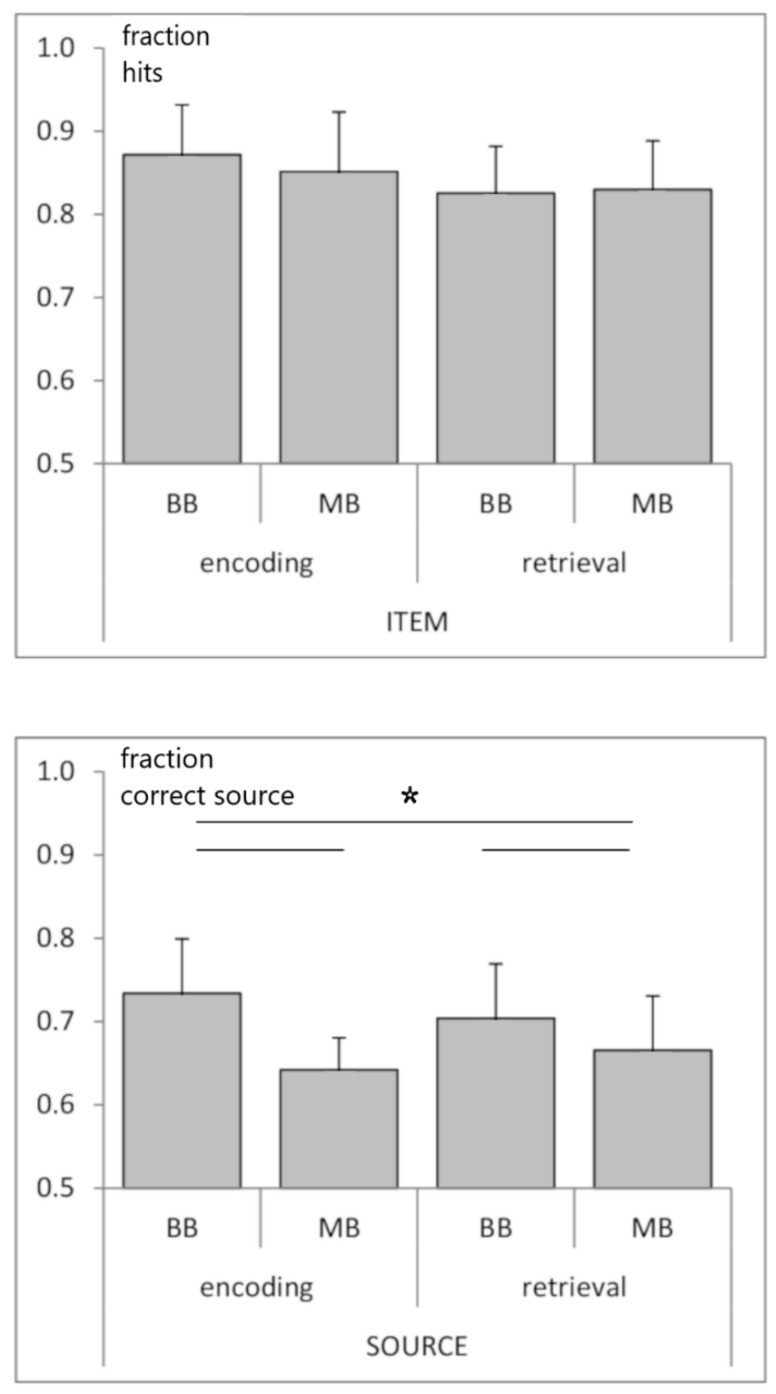
**Modulation of memory performance by beat stimulation:** fraction of hits (top, item memory) and fraction of correct source responses (bottom, source memory) for binaural (BB) vs. monaural (MB) beat stimulation during encoding and retrieval. Horizontal lines and asterisks indicate a significant ANOVA main effect of beat condition (but not of task phase) for source memory (*p* < 0.05).

A two-way repeated-measures ANOVA (factors: beat condition (binaural, monaural); task phase (encoding, retrieval)) revealed a main effect of beat condition for source memory (F_1,4_ = 8.095; *p* < 0.05; enhanced source memory for binaural vs. monaural) and no effects or interaction for item memory; Figure 2).

### 3.3. Relationship Between Beat Stimulation and Memory Performance: Neural Data

First, we identified neurons showing different firing rates for binaural vs. monaural beat stimulation (see Table 1). This analysis revealed 41 units for the encoding phase (23% of all units; 20 increase; 21 decrease), of these 30 on the left side (8 amygdala (26% of all amygdala units on the left side), 5 hippocampus (38%), 14 entorhinal cortex (29%) and 3 parahippocampal cortex (16%)) and 11 on the right side (4 amygdala (22%), 2 hippocampus (11%), 2 entorhinal cortex (14%) and 3 parahippocampal cortex (18%)). The contributions of the five participants to the 41 units were: p1 (7); p2 (12); p3 (2); p4 (18); p5 (2).

A total of 75 units were found for the retrieval phase (42% of all units; 32 increase; 43 decrease); of these, 52 were on the left side (18 amygdala (58% of all amygdala units on the left side), 6 hippocampus (46%), 17 entorhinal cortex (35%) and 11 parahippocampal cortex (58%)), and 23 were on the right side (5 amygdala (28%), 2 hippocampus (11%), 6 entorhinal cortex (43%) and 10 parahippocampal cortex (59%)). The contributions of the five participants to the 75 units were: p1 (16); p2 (29); p3 (5); p4 (22); p5 (3). Total numbers of beat-stimulation responsive neurons were significantly higher than expected by chance both, on the left (encoding: p_binom_ = 3 × 10^−14^; retrieval: p_binom_ = 3 × 10^−37^) and right side (encoding: p_binom_ = 5 × 10^−4^; retrieval: p_binom_ = 1 × 10^−13^; see Table 1 for region-specific significance values). As a trend, a larger proportion of neurons differentially responded to binaural vs. monaural stimulation on the left compared to the right side (chi-square-Tests; encoding: chi-square_1,N=180_ = 2.71; *p* = 0.100; retrieval: chi-square_1,N=180_ = 2.77; *p* = 0.096). Interestingly, there was no significant overlap between beat-stimulation responsive neurons during encoding and retrieval either on the left side (17 units; p_binom_ = 0.226), or on the right side (5 units; p_binom_ = 0.315).

**Figure 3:**
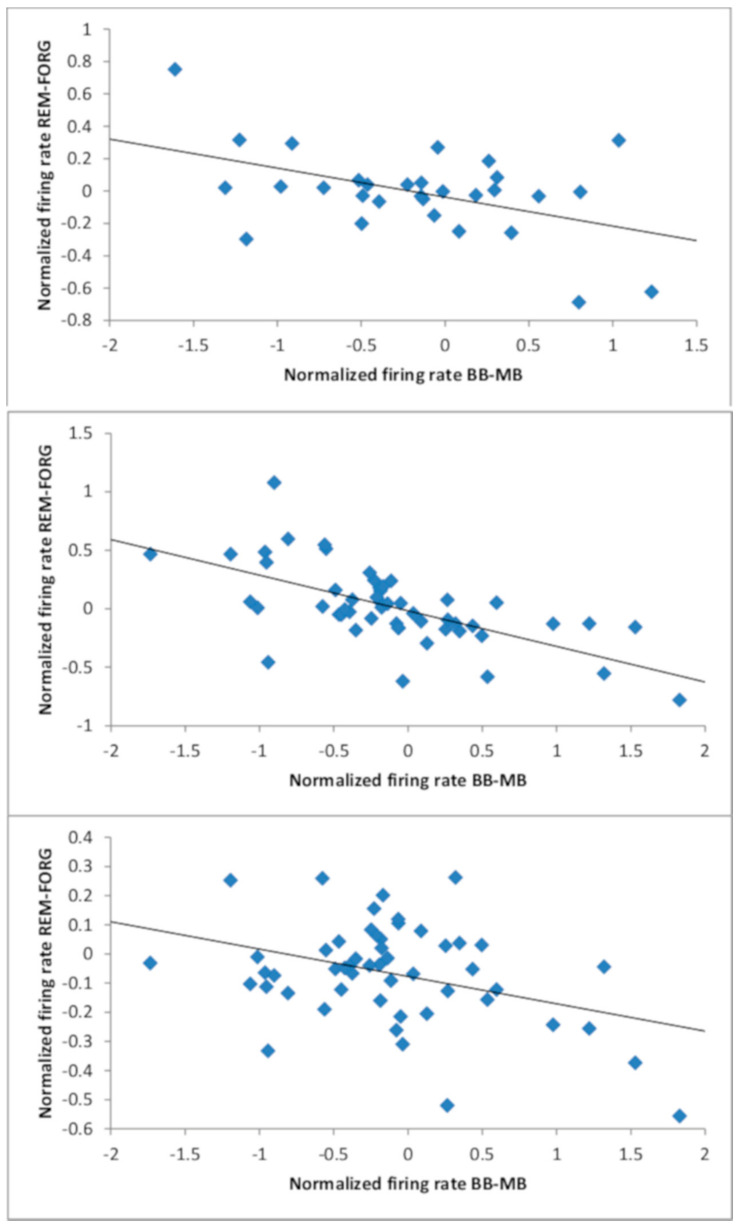
**Correlation between beat stimulation-related and memory-related firing rate differences on the left hemisphere:** Top: Encoding phase; item memory; 1500–2000 ms window; *r* = −0.47 (*p* = 0.009). Middle: Retrieval phase; item memory; 1000–1500 ms window; *r* = −0.64 (*p* = 4 × 10^−7^). Bottom: Retrieval phase; source memory; 1000–1500 ms window; *r* = −0.38 (*p* = 0.006).

We then calculated Pearson’s correlations between normalized beat stimulation-related and memory-related firing rate differences for these neurons. There were no statistically significant correlations for the right hemisphere. However, we found several significant negative correlations for the left hemisphere (see Table 2 and Figure 3). Numerically, all but one of the 16 correlation values for the left hemisphere were negative. During encoding, item memory-related firing rates were correlated with beat-related firing rates in the 1500–2000 ms window (*r* = −0.47, *p* = 0.009). During retrieval, item memory-related firing rates were correlated with beat-related firing rates in the 500–1000 ms and 1000–1500 ms windows (*r* = −0.49, *p* = 2 × 10^−4^ and *r* = −0.64, *p* = 4 × 10^−7^). The latter correlation even survived Bonferroni correction across all tests (*n* = 32) conducted for both hemispheres. Finally, source memory-related firing rates during retrieval were correlated with beat-related firing rates in the 1000–1500 ms window (*r* = −0.38, *p* = 0.006).

It is important to note that average firing rates (across units) do not reflect unit-specific beat-related effects, because unit-specific effects can either be positive or negative. For instance, the average firing rates for the beat-responsive units on the left side during the different conditions (binaural/monaural; encoding/retrieval) and time windows ranged from 2.97 Hz to 4.76 Hz. However, for each time window and both encoding and retrieval, paired *t*-tests (across units) between the binaural and monaural conditions were non-significant (each *p* > 0.05). This means that there was no general tendency (across units) for beat-related shifts of firing rates to occur in the positive or negative directions.

The observed negative correlations suggested that beat stimulation has an impact on memory via shifting baseline firing levels; therefore, we further aimed to explore this possibility. First, we performed an additional analysis for the beat-responsive units on the left side. We multiplied the firing rate differences for binaural minus monaural in the pre-stimulus window with the signs (+1 or −1) of the post-stimulus beat effects. For the resulting values, two-sided *t*-tests revealed highly significant differences from zero (encoding: *p* < 1 × 10^−7^; retrieval *p* < 1 × 10^−8^). This confirms that beat stimulation also had an impact on firing rates in the pre-stimulus window.

Secondly, we calculated Pearson’s correlations between normalized beat stimulation-related firing rate differences in the pre-stimulus window [−500 ms; 0 ms] and memory-related firing rate differences in the post-stimulus windows for the same neurons (see Table 3). Corresponding to the four cases of statistically significant negative correlations described above, three time windows again showed significant negative correlations. Moreover, this analysis revealed two additional cases of negative correlations. Beat stimulation-related firing rate differences in the pre-stimulus window were negatively correlated with memory-related firing rate differences in the 1000–1500 ms window for source encoding (*r* = −0.47, *p* = 0.008), as well as in the 0–500 ms window for item retrieval (*r* = −0.36, *p* = 0.010). These findings corroborate the idea that beat stimulation-related shifts of baseline firing levels mediate the effects on memory performance.

Moreover, we performed an additional analysis based on extracting memory-related differences only for the actual runs with beat stimulation (encoding/color and retrieval/scene runs). In line with the previous analyses, we observed a significant negative correlation between beat-related and item memory-related firing rates during retrieval in the 1000–1500 ms window (*r* = −0.38, *p* = 0.006). A negative correlation was also evident between beat-related baseline firing rates and item memory-related firing rates in this time window (*r* = −0.43, *p* = 0.002). Moreover, we found a positive correlation between beat-related and source memory-related firing rates during retrieval in the 0–500 ms window (*r* = 0.36, *p* = 0.009). However, there was no significant correlation (r = 0.31, *p* = 0.025) between beat-related baseline firing rates and source memory-related firing rates in this time window.

Finally, we conducted an exploratory additional analysis to investigate whether firing rate levels were directly related to remembered versus forgotten differences, as suggested by models of homeostatic plasticity. We calculated the following Pearson’s correlations across beat-responsive units (left side), separately for units with positive and units with negative remembered versus forgotten differences: (i) between pre-stimulus activity [−500; 0 ms] in the monaural or binaural condition and remembered versus forgotten differences in the post-stimulus windows; and (ii) between post-stimulus activity in the monaural or binaural condition and post-stimulus remembered versus forgotten differences. This approach yielded 2 (pos. diff./neg. diff.) × 2 (a/b) × 2 (monaural/binaural) × 4 (time windows) × 2 (item/source) × 2 (encoding/retrieval) = 128 correlation values. From these correlations, 20 had *p*-values between 0.05 and 0.1, and five had *p*-values below 0.05. Importantly, for the positive remembered versus forgotten differences, 62 out of 64 correlations were negative (two positive correlations: *p* > 0.65), and for the negative remembered versus forgotten differences, all 64 correlations were positive. Moreover, when calculating these correlations for the absolute values of remembered versus forgotten differences across all beat-responsive units (left side), 27 out of 64 correlations had *p*-values between 0.05 and 0.1, 13 had *p*-values below 0.05, and all correlations were negative. This indicates that higher levels of firing rates correspond to decreased memory effects and lower levels of firing rates correspond to increased memory effects. These data corroborate the interpretation of our findings in terms of homeostatic plasticity, because increased/decreased levels of firing rates impede/favor synaptic potentiation according to the BMC rule (Bienenstock et al., 1982).

## 4. Discussion

An impact of beat stimulation on memory performance has been described by several groups (for overviews, see, e.g., [1,2]). We have reported that binaural and monaural 5 Hz beat stimulation modulated memory performance in opposite directions based on data from a larger patient group [5]. Binaural beats were related to enhanced, and monaural beats to impaired item and source memory. In the subgroup analyzed here, only a significant behavioral effect on source memory was evident. In accordance with previous intracranial EEG findings [4,5], we observed a significant effect of binaural versus monaural beat stimulation on firing rates in a large fraction of neurons within the medial temporal lobe. As a trend, a larger proportion of neurons differentially responded to beat stimulation on the left versus right side. Similarly, another study reported that theta EEG responses to 6 Hz binaural beats were dominant in the left hemisphere [21].For the beat-stimulation responsive neurons, we detected statistically significant negative correlations between firing rate differences for binaural versus monaural beats and remembered versus forgotten items/associations in the left hemisphere. Importantly, such negative correlations were also observed between beat stimulation-related firing rate differences in the pre-stimulus window and memory-related firing rate differences in the post-stimulus windows. Therefore, we interpret our findings as indicating that beat stimulation is linked to memory performance via shifting baseline firing levels and not via directly adding to memory-related firing rate changes. Expressly speaking, we suggest that by shifting baseline levels, differences between memory-related and baseline firing rates are modulated, consistent with increased memory performance for binaural versus monaural beats.

The correlation values listed in Table 2 and Table 3 show a larger impact of beat stimulation on memory retrieval than encoding (six versus three significant correlations). This is to be expected because electrophysiological processes during retrieval are instantly related to memory performance, whereas electrophysiological processes during encoding are related to later memory performance (subsequent memory); thus, memory-related differences are typically less robust during encoding versus retrieval. For instance, in a previous study using the same dataset [9], we could statistically identify neurons related to successful versus unsuccessful memory retrieval, but not related to memory encoding. Moreover, of the nine significant correlations listed in Table 2 and Table 3, only one occurred in the earliest time window [0; 500 ms], and in this case (Table 3; item retrieval), the correlation in the later time windows [500; 1000 ms] and [1000; 1500 ms] showed much lower *p*-values. This is plausible because responses in the earliest time window mainly reflect stimulus-related/perceptual processing, while responses in the later time windows rather reflect memory-related processing (e.g., [9,22]).

In the larger participant group investigated previously [5], we had found a statistically significant behavioral effect of beat stimulation on both item and source memory. In the subgroup investigated here, we only observed a behavioral effect on source memory. However, correlations between beat- and memory-related firing rate differences (Table 2 and Table 3) were predominantly significant for item memory. One reason for these discrepancies may be the lower statistical power in the present study due to the small group size. Another possible reason is that for the present paradigm, single-unit responses related to item memory appeared stronger than responses related to source memory. In the previous study using the same dataset [9], we could identify 26 units related to successful versus unsuccessful item retrieval, but only 15 units related to source retrieval.

Our results are in accordance with the BCM rule of homeostatic plasticity [10], proposing that high levels of previous neuronal activity favor synaptic depression, and low levels favor facilitation. Experimental support for such an interrelation has, for instance, been reported in studies investigating the impact of light deprivation on visual processing in rats [23], as well as transcranial direct current stimulation on the learning of motor behavior [24] and visuomotor coordination in humans [25]. Moreover, a compensatory increase/decrease in synaptic strength after chemically or optogenetically induced inhibition/excitation of neural activity has also been shown in rodent hippocampal neurons [26,27]. Finally, the left-hemispheric localization of our findings is in line with its well-known specialization for language processing [12]. As mentioned before, a limitation of the current investigation is the small number of participants. Examining the impact of beat stimulation on memory in a larger number of epilepsy patients with implanted microwires may yield statistically more robust single-unit and behavioral results. Moreover, future studies with larger cohorts may disentangle region-specific effects within the medial temporal lobe, which was not possible in the current study. Another important future direction would be to study the interrelation between the effects of beat stimulation on firing rates and iEEG phases. Taken together, our results support the hypothesis that auditory beat stimulation has an impact on memory performance via the modulation of single neuron activity within the medial temporal lobe.

## Figures and Tables

**Table 1:** Overview of region-specific results of binomial tests.

				Entorhinal	Parahippocampal
		Amygdala	Hippocampus	Cortex	Cortex
**ENCODING**	**LEFT**	26% (1 × 10^−4^)	38% (3 × 10^−4^)	29% (8 × 10^−8^)	16% (n.s.)
	**RIGHT**	22% (0.011)	11% (n.s.)	14% (n.s.)	18% (n.s.)
**RETRIEVAL**	**LEFT**	58% (4 × 10^−16^)	46% (2 × 10^−5^)	35% (1 × 10^−10^)	58% (3 × 10^−10^)
	**RIGHT**	28% (0.002)	11% (n.s.)	43% (3 × 10^−5^)	59% (1 × 10^−9^)

Results of binomial tests assessing whether numbers of neurons showing different firing rates for binaural vs. monaural beat stimulation were significantly higher than expected by chance. *p*-values below 0.05 are considered statistically significant. The proportions (%) of neurons with respect to the total number of neurons in each region are listed in parentheses.

**Table 2:** Overview of Pearson’s correlations between beat stimulation-related (BB-MB) and memory-related (REM-FORG) firing rate differences in the post-stimulus windows.

		TIME WINDOW			
		0–500	500–1000	1000–1500	1500–2000
**ENCODING**	**item**	n.s.	n.s.	n.s.	−0.47 (0.009)
	**source**	n.s.	n.s.	n.s.	n.s.
**RETRIEVAL**	**item**	n.s.	−0.49 (2 × 10^−4^)	−0.64 (4 × 10^−7^)	n.s.
	**source**	n.s.	n.s.	−0.38 (0.006)	n.s.

Correlation values for the left hemisphere are listed. Only correlations with *p*-values below 0.0125 (Bonferroni-correction for 4 time windows) are considered statistically significant.

**Table 3:** Overview of Pearson’s correlations between beat stimulation-related (BB-MB) firing rate differences in the pre-stimulus window [−500; 0 ms] and memory-related (REM-FORG) firing rate differences in the post-stimulus windows.

		TIME WINDOW			
		0–500	500–1000	1000–1500	1500–2000
**ENCODING**	**item**	n.s.	n.s.	n.s.	−0.47 (0.009)
	**source**	n.s.	n.s.	−0.47 (0.008)	n.s.
**RETRIEVAL**	**item**	−0.36 (0.010)	−0.48 (3 × 10^−4^)	−0.67 (4 × 10^−8^)	n.s.
	**source**	n.s.	n.s.	n.s.	n.s.

Correlation values for the left hemisphere are listed. Only correlations with *p*-values below 0.0125 (Bonferroni correction for 4 time windows) are considered statistically significant.

## Data Availability

In accordance with the ethics approval given by the ethics committee of the Medical Faculty of the University of Bonn and the guidelines of the German Research Foundation, pooled spiking data and program codes have been made publicly available to researchers on the Github Online Repository (https://github.com/LeilaChaieb/Fell_Workspace (accessed on 3 December 2020)).

## Supplementary Materials

The following are available online at https://www.mdpi.com/2076-3425/11/3/364/s1, Figure S1: Example of beat-responsive single-unit from amygdala, Figure S2: Example of beat-responsive multi-unit from entorhinal cortex, Figure S3: Examples of artifacts.
