# Supplementary material for: Auditory Beat Stimulation Modulates Memory-Related Single-Neuron Activity in the Human Medial Temporal Lobe
Source: Brain Sci. 2021 Mar 12;11(3):364. doi: 10.3390/brainsci11030364 (PMC8000797; doi:10.3390/brainsci11030364)
Supplement: Supplementary file 1 [file brainsci-11-00364-s001.pdf]

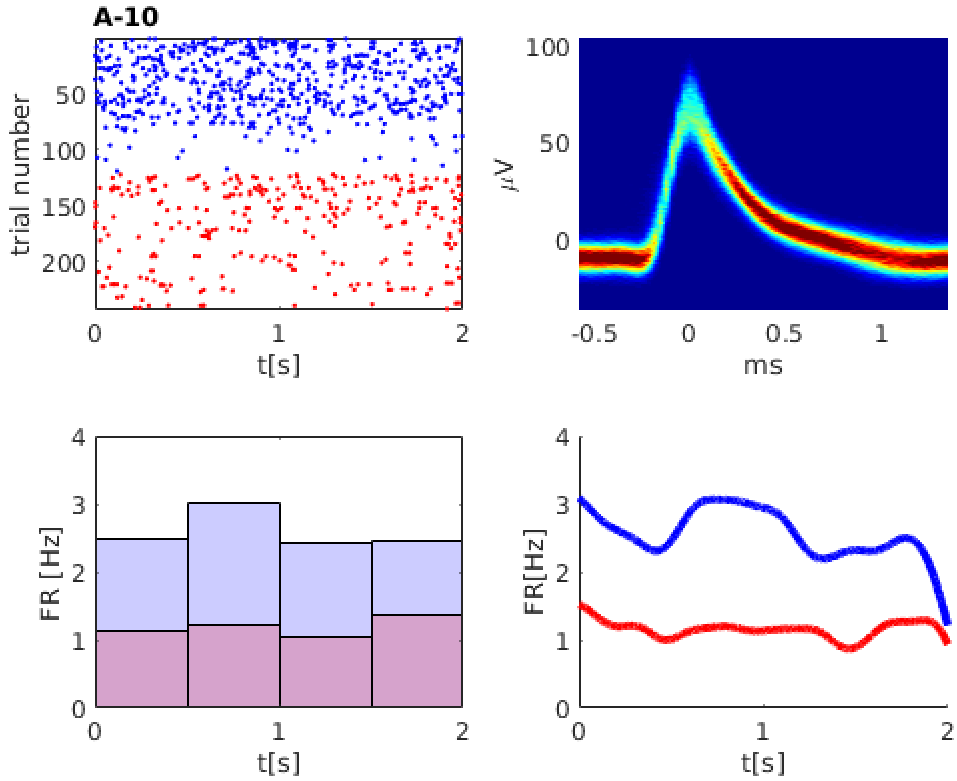

Scheme 1. Example of beat-responsive single-unit from amygdala. Top left: raster plots of spike times relative to stimulus onset for binaural beat stimulation (blue) and monaural beat stimulation (red). Top right: Density plots of all spike waveforms. The plots show 2-dimensional histograms of spike voltages over time. The color code depicts the percentage of spikes (denominator: all spikes recorded for this unit) with the specified voltage at the given time point. Bottom left: Histograms of average firing rates within consecutive 500 ms windows (stimulus onset at t=0; binaural: blue; overlap binaural and monaural: purple). Bottom right: Histograms of continuous average firing rates (stimulus onset at t=0; binaural: blue; monaural: red). Firing rates were calculated within 1ms time intervals and were subsequently convolved with a Gaussian kernel (standard deviation: 100 ms).

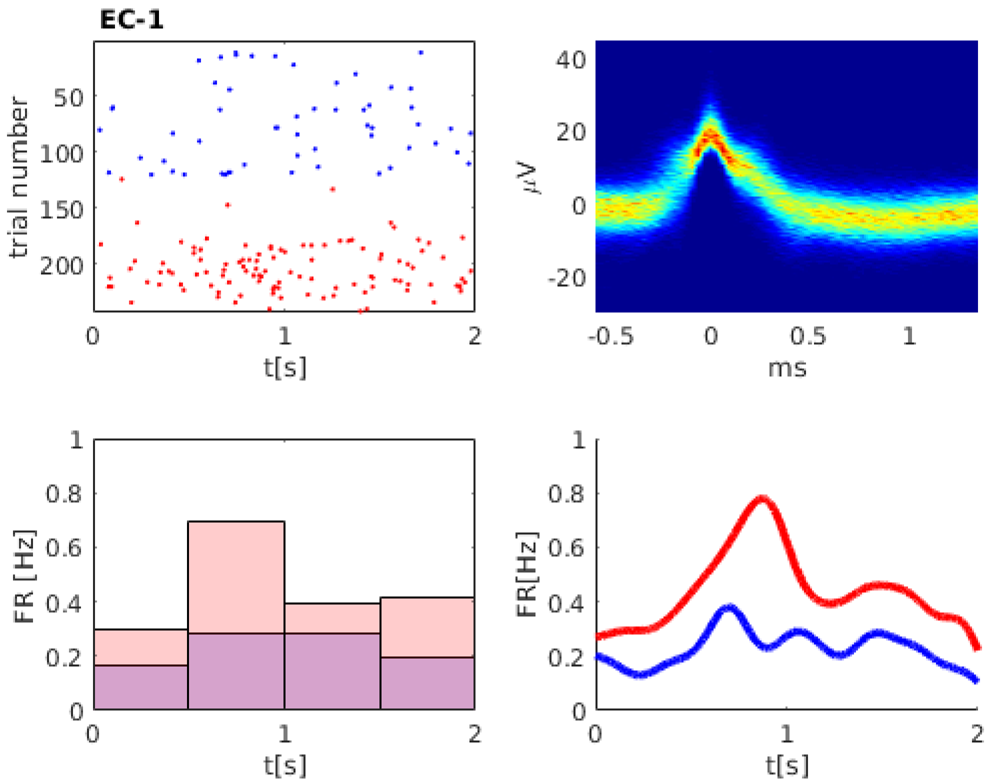

Scheme 2. Example of beat-responsive multi-unit from entorhinal cortex. Top left: raster plots of spike times relative to stimulus onset for binaural beat stimulation (blue) and monaural beat stimulation (red). Top right: Density plots of all spike waveforms. The plots show 2-dimensional histograms of spike voltages over time. The color code depicts the percentage of spikes (denominator: all spikes recorded for this unit) with the specified voltage at the given time point. Bottom left: Histograms of average firing rates within consecutive 500 ms windows (stimulus onset at t=0; monaural: pink; overlap binaural and monaural: purple). Bottom right: Histograms of continuous average firing rates (stimulus onset at t=0; binaural: blue; monaural: red). Firing rates were calculated within 1ms time intervals and were subsequently convolved with a Gaussian kernel (standard deviation: 100 ms).

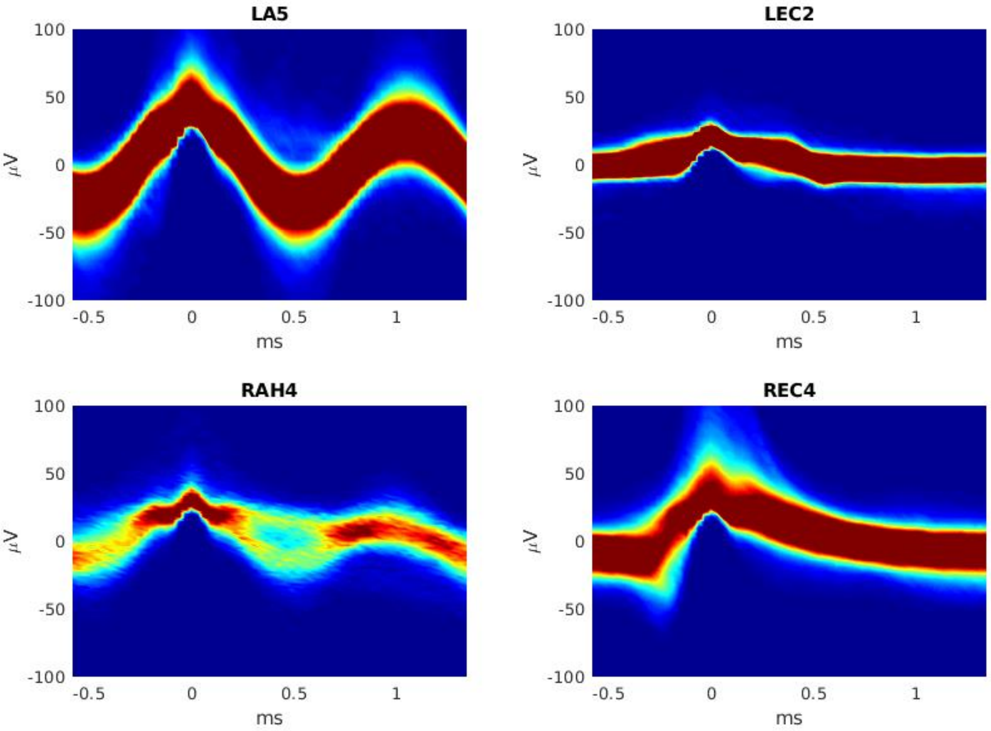

Scheme 3. Examples of artifacts. Density plots of waveforms classified as artifacts. The plots show 2-dimensional histograms of spike voltages over time. The color code depicts the percentage of spikes (denominator: all spikes recorded for this unit) with the specified voltage at the given time point.
